# Is it really better to “die” than “live”? Reflections on the practice of “death with dignity” in China

**DOI:** 10.3389/fpubh.2024.1426257

**Published:** 2024-08-14

**Authors:** Long Chen, Minghui Ren

**Affiliations:** Department of Global Health, School of Public Health, Peking University, Beijing, China

**Keywords:** death with dignity, living will, medical autonomy, hospice care, palliative care

## Abstract

**Aim:**

To analyze the causes of the controversy caused by “death with dignity” in China, and to provide some useful thoughts for the practical exploration of “death with dignity.”

**Subject and methods:**

By combing the periodical literature, legal texts and practice, we find that the Medical Regulations of Shenzhen Special Economic Zone, which was revised and passed by China in 2022, recognized the legal effect of “living will” for the first time in legislation, which triggered a wide-ranging social discussion on “death with dignity” and brought many controversies.

**Results:**

Due to the influence of traditional culture, policies and laws, medical service supply capacity and other factors, death with dignity suffers from great practical resistance.

**Conclusion:**

The exploration of “death with dignity” system needs to start with the problems encountered in practice, focusing on cultivating a good system implementation environment, strengthening the top-level design of “death with dignity” system, and improving the national social security system for hospice care.

## 1 Introduction

With the continuous improvement of people’s material and cultural life, ordinary citizens have begun to pay more and more attention to the right to die while attaching importance to the right to life. In the process of citizens’ struggle for the right to “die,” a right to die with dignity was put forward (hereinafter referred to as death with dignity). Although there are still controversies about the concept and connotation of death with dignity in academic circles at present, it is generally believed that death with dignity means that patients give up some unnecessary life-prolonging medical measures (such as intubation, dialysis, etc.) when they enter the end of their lives, so as to alleviate the physical and mental torture caused by diseases and let their lives inherit the simple view of nature and meet their death with dignity (1). In theory, people often equate death with dignity with euthanasia (2), but in fact, there is a big difference between them. “Euthanasia” generally refers to the act of taking certain measures to end the patient’s life in advance for the purpose of alleviating the patient’s pain when the patient is suffering from an incurable disease and is on the verge of death (3). However, death with dignity is different from euthanasia. Death with dignity gave up some unnecessary medical care measures to prolong life under the condition of respecting patients’ wishes. It is not like euthanasia, where the actor takes active measures to end patients’ lives ahead of schedule (4). In addition, it should also be noted that the concept of death with dignity used in this paper should also be distinguished from the “ethnic cleansing” movements such as sterilization, euthanasia and mass slaughter forced by the Nazis during World War II. The death with dignity referred to in this paper was practiced on the basis of following the true feelings of patients, rather than being forced by outside powers. Moreover, the application premise of death with dignity in this paper is that the patients at the end of life have signed a legal and effective living will (5). The so-called living will was first seen in a paper published in the Indiana Law Journal in 1969 (6). The author believes that since the law has allowed people to make the entrustment about property distribution in advance when they are conscious, then people should also have the right to make and sign the entrustment about medical choice in advance. The living will can be regarded as an advance medical instruction of the patient, which specifically refers to a series of instructions and documents signed by the patient when the state of consciousness is clear and true, aiming at explaining what kind of medical care is “necessary” or “not” at the end of incurable life, such as whether to receive surgical chemotherapy and intubation medical services. A living will is similar to a legal document of a will or trust (7), but this “will” is special, and it deals not with personal property rights, but with personal life rights.

With the official promulgation of the revised Medical Regulations of Shenzhen Special Economic Zone in 2022, China has recognized the legal effect of living wills for the first time in legislation, and the social discussion about “death with dignity” in academic circles has gradually increased, which has also brought many debates (8). Therefore, after analyzing the reasons behind the controversy caused by death with dignity, this paper puts forward some suggestions based on the reality, so as to provide some useful thoughts for the system construction in death with dignity.

## 2 The controversy caused by death with dignity

Since the concept of death with dignity was put forward, it has attracted great attention from people from all walks of life, such as medicine, bioethics, law and so on, and it has also been debated in theory, among which two completely different academic viewpoints and positions have been formed around the legitimacy of death with dignity.

### 2.1 Position 1:“death with dignity” respect patients’ medical autonomy

The supporters believe that “death with dignity” is a manifestation of respecting patients’ “right to decide independently,” which is conducive to maintaining the personal dignity of patients at the end of their lives and a concentrated expression of respecting patients’ medical autonomy (9). In 1976, the California Natural Death Act was promulgated, which was the first draft legislation on death with dignity in the world. The bill allows citizens to sign an effective “living will” before their death to dispose of their core life rights and interests (10). Subsequently, Canada, France, Japan, South Korea, and Singapore have also promulgated special laws and regulations to promote domestic death with dignity legislation (11). In today’s increasingly aging society, it has become a universal realistic demand for the development of the country and society to try to provide the older adult with high-quality and equal medical services to the greatest extent. In the process of promoting the development of hospice care for the older adult, it is particularly important to really pay attention to the physical and mental health needs of the older adult, respect their inner wishes and help them realize their inner demands. The starting point of “death with dignity” is to take patients at the end of life as the center, and to realize the protection, respect and guarantee at the end of life by responding to their inner demands.

### 2.2 Position 2: “death with dignity” challenges patients’ life rights and personal dignity

Another scholar believes that the patient’s “medical autonomy” is difficult to measure, and it is more difficult in clinical operation. Do patients have the ability of rational analysis, judgment and decision-making to give up their prudent life interests under the condition of patients’ lack of full and necessary understanding of their own life and health status and serious asymmetry of medical information (12)? If innocent patients are driven by the illegal purpose of a third party in this process, they will be easily used by others as “tools” to harm themselves. In addition, should the wishes of the patient’s family members and attending doctors be respected when the patient makes a decision on whether to accept or not to accept medical services? When there is a conflict between the two, how should we make an appropriate decision and plan (13)? Because of the above disputes, the clinical practice of death with dignity will also face severe challenges.

## 3 Dilemma in the practice of “death with dignity”

### 3.1 It seems to be in contradiction with the values of Chinese traditional culture

According to traditional culture of China, “Among all the virtues, filial piety comes first” and “Filial piety,” as a general term for respecting relatives and the older adult, is highly respected by the traditional China society. The Analects of Confucius says: “A disciple is filial when he enters, and a younger brother when he leaves.” “It’s very rare that filial and fraternal men would offend against their superiors. It’s unheard of that men who do not want to offend against their superiors would stir up trouble.” “Those who are filial are the foundation of benevolence.” For Confucianism, which advocates benevolence and morality, “filial piety” is placed in a higher position, which can be extended from consanguineous family ethics to general social relations and become the basic criterion for dealing with personal social relations. The Book of Rites, The Doctrine of the Mean, says, “A benevolent person is a human being, and a kiss is the greatest.” Confucius believes that “benevolence” is the highest criterion of individual behavior, and as a universal principle, it should be fundamentally followed in practice. To practice this principle, we must start from the people and things around us. This is “filial piety.” Mencius further improved the philosophical basis of “filial piety.” Mencius advocated the “five ethics” relationship criterion of loyalty, filial piety, filial piety, forbearance and kindness, and regarded “filial piety” as the core of ethics and morality, ranking first in the “five ethics.” “Mencius told the son” said: “The way of Yao and Shun is just filial piety.” For Mencius, who admired Saint Wang Zhidao, “filial piety” was the highest expression of personal virtue.

Thus, in a society infiltrated by Confucian culture for a long time, “filial piety culture” does matter in decision-making scenarios. How to properly handle the relationship between parents and children and brothers and sisters has become an important pole in building a harmonious family relationship. Under the soil of China’s traditional culture, the “five ethics” relationship criterion with “filial piety” as the core has become the basic morality and behavior criterion for regulating social communication between people. As Fei Xiaotong wrote in Earthbound China, “Combination of etiquette and law” is an important feature of traditional society in China (14), Out of the “etiquette” into the “punishment.” From “Zhou Gong made rites” in the Western Zhou Dynasty to “The Five Degrees of Mourning Clothing System” in the Jin Dynasty and then to “ten evils” felony in the Sui and Tang Dynasties. The “etiquette” in the society was deeply embedded in the national legal system, which violated social conventions. “The Analects of Confucius “says: "That parents, when alive, be served according to propriety; that, when dead, they should be buried according to propriety; and that they should be sacrificed to according to propriety.” Whether parents are alive or dead, they should be filial in accordance with the “ritual” rules followed by society. Talking about his death before his parents died, and giving up or terminating the treatment of his the relatives’ physical illness when they have not completely lost vital life signs, is unacceptable and inappropriate in China’s traditional society, which values ceremony and filial piety. Therefore, considering the contradiction and conflict between the value concept behind “death with dignity” and Chinese traditional ethical culture, it will inevitably encounter great practical resistance when the system “falls to the ground.” Moreover, the implementation of the “death with dignity” system in China at this stage will also face challenges such as the absence of policies and regulations, insufficient material supply, deficit in revenue and expenditure, shortage of professionals, inadequate occupational management guarantee and low social support. This has also caused difficulties in the operation of death with dignity in practice.

### 3.2 The operational procedures of death with dignity have yet to be solved

On June 23, 2022, the newly revised Medical Regulations of Shenzhen Special Economic Zone was officially promulgated, and China recognized the legal effect of death with dignity (living will) for the first time in legislation. Among them, Article 78 of the Medical Regulations simply stipulates the content and form of death with dignity. [See Article 78 of the Medical Regulations of Shenzhen Special Economic Zone: If a patient or his close relatives provide a patient with the following conditions, the medical institution shall respect the intention of the patient’s living will when implementing medical measures at the end of the incurable injury or at the end of his life: (1) There is a clear intention of taking or not taking traumatic rescue measures such as intubation and cardiopulmonary resuscitation, using or not using a life support system, and continuing treatment of the primary disease; (2) notarized or witnessed by two or more witnesses, and the witnesses shall not be medical and health personnel involved in the treatment of patients; (3) in writing or audio-visual recording, unless notarized, in writing, it shall be signed by the testator and witnesses and indicate the time; In the case of audio and video recording, the names or portraits of the testator and witnesses and the time shall be recorded].

Obviously, Article 78 of the Medical Regulations is only a principled provision for death with dignity, and the specific operating procedures are not complete. For example, how to judge whether the patient is in the “incurable injury terminal” or “dying” stage, and how to determine the clinical standard? Should death with dignity give due consideration to the opinions of his close relatives when signing? Can the patient entrust a third person to handle it beforehand? Wait a minute. All the above problems need to be further improved by legislation.

### 3.3 The supply of hospice care services is prominent

For patients who implement death with dignity, it is not to completely give up and stop all the clinical diagnosis and treatment measures, but to give up some unnecessary life-prolonging treatment measures and adopt a more “humanized” medical care plan for patients with full respect for their inner wishes, which is called hospice care in academic circles (15). “Hospice care” is a kind of more “warm-hearted” care, which requires hospitals to provide patients with general diagnosis and treatment services and pay more attention to the improvement of medical service quality, which also puts forward higher requirements for the hospital’s medical service level. In October, 2022, the state issued the 2022 National Bulletin on the Development of Aging (hereinafter referred to as the Bulletin), which showed that by the end of 2022, there were 280.04 million older adult people aged 60 and above in China, an increase of more than 10 million over the previous year, accounting for 19.8% of the total population. There are 387,000 institutions and facilities for the aged in China, with a total of 8.294 million beds for the aged (16). According to a little statistics, single institutions and facilities for the aged need to serve more than 700 older adult people, and single beds for the aged need to serve more than 30 older adult people. With the increase of the total number of older adult people in China year by year and the deepening of aging, the demand for hospice care services will also increase accordingly. However, compared with the huge older adult population in China, the supply capacity of hospice care services in China is insufficient, and the contradiction between supply and demand is gradually prominent.

## 4 The perfect path of death with dignity system

At present, death with dignity’s system exploration needs to start with the problems encountered in practice, focusing on cultivating a good system implementation environment, strengthening the top-level design of death with dignity system, and improving the national social security system for hospice care, so as to better safeguard and safeguard the basic rights and interests of patients at the end of their lives.

### 4.1 Cultivate a good environment for system implementation

At this stage, China is in the initial stage of death with dignity system exploration, and the landing of a new social system needs the support of a good social environment. Based on a survey on the knowledge, attitude and behavior of 260 older adult inpatients in a third-class first-class hospital in Wuhan, it shows that the knowledge, attitude and behavior of older adult patients about living wills are medium, and the level of knowledge dimension is the lowest (17). In order to change this situation, it is necessary for the administrative, educational, medical, news media and other departments to properly carry out bioethics publicity and education for the public, publicize the values of sacredness and quality of life, and advocate a brand-new view of life and death. Only when “top-down” institutional innovation and “bottom-up” interest drive interact with each other, and a two-way interaction and feedback mechanism is established, can the organic integration of system and reality be realized.

### 4.2 Strengthen the top-level design of death with dignity system

Although the Medical Regulations of Shenzhen Special Economic Zone recognizes the legal effect of death with dignity for the first time in legislation, there are some vague concepts and criteria for judging patients’ corresponding behavior ability in the existing regulations. The range of medical measures that patients can choose in their living wills is unclear; the subject of responsibility and the unclear legal liability provisions (18). In order to change this situation, the state needs to strengthen the top-level design of relevant policies and regulations in death with dignity and improve the specific operating procedures implemented in death with dignity. Specifically, considering that the rights and interests disposed by death with dignity are the core life rights and interests of individuals (19), reasonable restrictions should be made on the application subjects. The applicant must be a natural person with full capacity for civil conduct, and the applicable standards can refer to the provisions of Articles 17 and 18 of the Civil Code. (See Article 17 of the Civil Code: A natural person over the age of 18 is an adult. Natural persons under the age of eighteen are minors. Article 18: Adults are persons with full capacity for civil conduct and can independently carry out civil juristic acts. Minors over the age of 16 who take their own labor income as their main source of livelihood are regarded as persons with full capacity for civil conduct.)

When it is necessary by law (such as falling into consciousness disorder), a medical agent can be entrusted, but the patient’s written entrustment documents must be produced and the best interests of the patient should be followed as the basic principle. In the form of application, in order to show respect for the medical autonomy of patients at the end of life, but also to show a cautious attitude toward the disposition of life rights and interests, so that every patient at the end of life can be cautious when deciding on matters related to personal life and health. In principle, the application submitted by the applicant should be mainly in writing. In terms of witnesses, in comparative law, there is national legislation that requires at least two parties with full civil capacity to witness on the spot, and at least one of them is a doctor (20). China’s legislation can take it as a reference, and it is stipulated that the signing and application process of death with dignity should be witnessed by at least two adult parties. At the same time, in order to prevent the related ethical and moral risks, the witness should not be the spouse, close relatives, heirs and other interested subjects of the patient. In terms of application content, death with dignity is a document of “want” or “do not want” medical care measures signed by patients at the end of their lives, and its content must reflect the true feelings of patients. If they go back on their word after applying for personnel, they can change or cancel their medical care instructions at any time, and they will not be treated unfairly because of the change or cancelation. In terms of filing and examination, in order to ensure the legitimate and reasonable exercise of death with dignity, the implementation of death with dignity needs to be filed and examined with a notary office. The state should set up a special death with dignity implementation supervision institution (or entrust relevant organs or social welfare organizations to supervise the implementation) as an independent third party with no interest to supervise the social implementation in death with dignity. In terms of the composition of the regulatory body, in order to ensure the scientific nature of the supervision decision, its members should accommodate experts and scholars from different disciplines such as medicine, ethics and law, and the regulatory body should improve its internal rules and regulations, regularly publish the list of members, and accept the supervision of the general public. In terms of relevant subject responsibilities, the enforcement applicant in death with dignity should be a patient at the end of his life. In order to avoid the risks between doctors and patients in the implementation, if the hospital legally and reasonably implements the death with dignity Agreement according to the contract, it does not need to bear the adverse legal consequences. On the other hand, if the hospital violates the contract or improperly performs the agreement and infringes on the personal or property rights of patients, it should bear corresponding legal responsibilities.

### 4.3 Improve the national social security system for hospice care

With the increasing aging population in China, it is necessary to improve the social security system of hospice care in China. At this stage, China should clarify the admission, care and operation standards of hospice care, strengthen the internal management of medical staff and improve the supply capacity of hospice care services. The government should increase the financial expenditure of medical services, strengthen the echelon and discipline construction of medical talents, and establish the norms of hospice care occupation, training and clinical practice. In discipline construction, medical curriculum should break the professional barriers between medicine and humanities and social sciences, and promote the deep cross-integration between medicine and other disciplines. In the distribution of medical resources, the management department should establish a fair and reasonable incentive mechanism, actively promote the fair distribution of medical resources between regions and between urban and rural areas, and realize the efficient allocation of medical human resources. In addition, hospice care service is an important social security measure. With the aging of our population, in order to improve the ability and security level of the whole society, enhance the sense of acquisition and happiness of the older adult, and better realize the protection of the rights and interests of the older adult, the state can try to bring hospice care into the social compulsory medical insurance to meet the increasing demand for hospice care (21).

## 5 Conclusion

Death with dignity is a brand-new outlook on life and death, full of humanitarianism and strong humanistic concern. Due to the influence of traditional culture, policies and laws, medical service supply and other factors, there is great practical resistance to social implementation in death with dignity. Therefore, focusing on cultivating a good system implementation environment, strengthening the top-level design of death with dignity system, and improving the national social security system for hospice care, so as to better promote the development of hospice care in China.

## Data availability statement

The original contributions presented in the study are included in the article/supplementary material, further inquiries can be directed to the corresponding author.

## Author contributions

LC: Conceptualization, Writing – original draft, Writing – review & editing. MR: Supervision, Validation, Writing – original draft.
